# Resilience mediates the influence of hope, optimism, social support, and stress on anxiety severity among Chinese patients with cervical spondylosis

**DOI:** 10.3389/fpsyt.2022.997541

**Published:** 2022-09-23

**Authors:** Yuying Chu, Yuqiang Zhang, Suyan Wang, Hongliang Dai

**Affiliations:** ^1^School of Nursing, Jinzhou Medical University, Jinzhou, Liaoning, China; ^2^The First Affiliated Hospital, Jinzhou Medical University, Jinzhou, Liaoning, China; ^3^Centre for Mental Health Guidance, Jinzhou Medical University, Jinzhou, Liaoning, China

**Keywords:** cervical spondylosis, anxiety, resilience, optimism, hope, perceived social support

## Abstract

**Objective:**

Cervical spondylosis (CS) is a potential stressor threatening mental health among affected individuals. This study was to analyze resilience level and associated factors among cervical spondylosis (CS) patients, and to explore the underlying mechanism of anxiety based on resilience-focused psychological variables.

**Methods:**

Resilience Scale-14 (RS-14), Zung Self-Rating Anxiety Scale (SAS), Herth Hope Index (HHI), Revised Life Orientation Test (LOT-R), Multidimensional Scale of Perceived Social Support (MSPSS), Perceived Stress Scale-10 (PSS-10) were used in this cross-sectional investigation among 250 CS patients.

**Results:**

The score of resilience was 65.58 ± 16.14. Hierarchical linear regression analysis revealed that hope, optimism, perceived social support, perceived stress, and whether having comorbid chronic diseases were the independent associates of resilience among CS patients, which explained 63.9% of the total variance. The structural equation model showed that hope, optimism, perceived social support and perceived stress affected anxiety *via* resilience, and hope and optimism also had direct effects on anxiety.

**Conclusion:**

Chinese patients with CS had moderate level of mental resilience, which was independently related to hope, optimism, perceived social support, perceived stress, and whether having comorbid chronic diseases. Resilience played a mediating role between various psychological variables and anxiety. Improving the level of resilience, hope, optimism and perceived social support and reducing the level of perceived stress are important strategies to reduce anxiety level. Relevant healthcare professionals should put more focus on the mental problems of Chinese CS patients and help maintain good psychological status by improving their resilience and associated psychological variables thereof.

## Introduction

Emerging evidence has revealed that people with chronic illness or worse health condition present greater anxiety when compared with healthy individuals (1). Cervical spondylosis (CS) refers a clinical manifestation characterized by cervical disc herniation and adjacent soft tissue lesions due to compression of spinal cord, nerves, and vertebral arteries (2). Early symptoms of CS are mainly neck and shoulder pain, and later symptoms are characterized by permanent nerve damage, including neck pain and movement weakness (3). In recent years, the incidence of CS has increased (4). A cross-sectional study published in 2016 showed that 35.9% of Chinese people are prone to chronic body pain such as head, neck, and shoulder (5). In another cross-sectional study published in 2018, the prevalence of chronic pain among Chinese older adults was as high as 49.8%, with legs/feet (25.5%), back (23.2%), and neck/shoulder (14.6%) being the most affected body parts (6). Notably, patients with CS not only experience physical pain, but also suffer from serious mental stress. Long course and recurrent symptoms of CS can easily cause depression, anxiety, and other emotional disorders among the affected individuals, and these negative emotions lead to low cognitive function, sleep disorders and poor quality of life (7). Unfortunately, the common treatment of CS focuses on the elimination or alleviation of physical symptoms, neglecting concomitant psychological disorders. Previous studies suggested that individuals with chronic back and neck pain have poorer flourishing mental health, as reflected by declined emotional, psychological, and social well-being, than those with other chronic conditions (8). Thus, more attention should be paid to psychological aspects of CS patients.

Intriguingly, individuals differ widely when they are confronted with the same adversity or stressors. Resilience represents the benign adaptive ability of individuals to face major stress events such as life adversity, external danger, physical and mental trauma (9). The level of resilience plays a big role in mental health (10, 11). A high level of resilience can fight negative emotions like anxiety and depression and benefits mental health. Low-resilience individuals have difficulty controlling negative emotions during stress and trauma, which can seriously affect their quality of life. Resilience intervention would thus potentially enhance patients' ability to cope with stress, reduce negative emotions, and improve their quality of life. Mounting studies suggested that low-resilience was critically related to diverse psychological conditions, such as anxiety (12–14). Notably, recent studies have proposed resilience as a new and more efficient solution to the maintenance of a good life and mental state in patients with chronic pain (15, 16). Surprisingly, however, the resilience level and related factors has never been explored among CS patients up to present.

Individual psychological qualities and social environment have been regarded as critical factors in building of resilience when in face of a certain adversity (15, 17). Among other psychological qualities, dispositional optimism and hope are two potentially important predictors for resilience in clinical or non-clinical samples, including those with chronic pain (13, 18, 19). Optimism, as a dispositional construct, refers to a tendency to consistently hold positive expectations for the future consequences, even confronting with uncertainty and adversity (20). As an important psychological resource, optimism has been increasingly found to be associated with more positive affect and lower negative psychological expressions, such as anxiety, depression, and post-traumatic symptoms (21–24). Optimistic individuals tend to recast an inherently unpleasant difficulty into a “blessing in disguise,” making them experiencing higher psychological and mental well-being during challenging times. For example, optimism has been characterized a positive mechanism in reducing rate of pain intensity and depressive symptoms in patients having rheumatoid arthritis (25). In addition, a recent study also showed that among women with fertility problems, those with higher trait optimism experienced significantly less negative perception and emotion during the stressful COVID-19 pandemic (26). Hope, as one of the prominent components of psychological capital, is regarded as a cognitive motivational component for attaining self-set goals (27). Hope has been found to be closely associated with positive coping strategies and negatively with negative affect status amid stressful situation (28). In line with that, studies also confirmed that hope help taper off posttraumatic stress disorder (PTSD) symptoms in patients with chronic diseases, such as hematological malignancies, oral cancer, or chronic obstructive pulmonary disease (13, 29). Hope has also been identified as protective factors against depressive symptoms among college students surrounding COVID-19 outbreak (30). Despite the positive and conducive effect of optimism and hope in maintenance of psychological well-being in people in face of diverse adversities, their correlation with resilience among CS patients was lacking, although existing literature showed that optimistic individuals or those with dispositional hope appear more resilient when addressing negative events, such as oral cancer and COVID-19 outbreak (13, 31, 32).

Stress and social support are two salient social environment factors influencing individual well-being. Stress refers to a complicated psychological state reflecting a series of physiological and behavioral processed when an actual or perceived threat occurs to disrupt equilibrium or homeostasis (33). CS patients are frequently experiencing multiple stressors, including pain, disabilities, and impaired quality of life (34–36), and thus, CS can be regarded as a potential stressful event for the affected individuals. Consistently, CS patients often had psychological comorbidities, such as depression and anxiety. Actually, in other clinical diseases or negative events, perceived stress also accounts for a range of unpleasant psychological responses, such as low resilience and PTSD symptoms in oral cancer patients (13, 33). Resilience was shown a mediator between stress and anxiety in general youth and nursing students during the COVID-19 pandemic (37, 38). Social support is a construct defined as the emotional, instrumental, tangible, and informational resources arising from social networks, or perceived love, care, esteem, and approval by others (39). Unlike perceived stress, social support was negatively associated with a plethora of negative psychological traits or states, such as anxiety, depression, and loneliness, etc., (22, 39, 40). Indeed a recent study has revealed that a low level of social support represents one of the significant predictors for anxiety in pregnant women receiving a high risk screening result for Down syndrome (41). Similarly, social support was shown to be negatively correlated with the level of depression and anxiety among Chinese medical students and Polish adults aged 30~44 years during the COVID-19 lockdown (42, 43). In addition, it was shown that social support was closely and negatively associated with anxiety and depression triggered by COVID-19, and positively with posttraumatic growth from this difficult condition (44). Higher levels of resilience and social support were associated with lower levels of anxiety and depression. Previous studies have confirmed that social support indirectly affected the levels of anxiety and depression *via* the mediating effect of resilience among lung cancer patients (45). Although stress and social support have shown to be critical determinants for modulation of psychological distress, whether and how these two variables affect CS patients is unclear.

According to the comprehensive analysis above, we herein aimed to fulfill the following three tasks: (1) Identifying the resilience level and its potential predictive factors, including above-mentioned psychological factors (i.e., hope, optimism, perceived social support, and perceived stress) among CS patients; (2) Considering that anxiety is a most common negative psychological state in CS patients (46), we also evaluated the dyadic and comprehensive relation between anxiety and other psychological variables; (3) Testing whether resilience played a mediating role in the association between other psychological variables and anxiety. We hope that our results can help improve mental health interventions in patients with CS *via* identifying the correlates of anxiety, especially resilience and its related factors.

## Materials and methods

### Participants

The subjects were 250 CS patients from a tertiary hospital in Jinzhou, China from February to September, 2021. The age of the participants ranged from 18 to 88 years, with average age being 44.04 ± 15.16 years. One hundred twenty-one (48.4%) participants were males and 129 (51.6%) were females. The purpose, significance and filling requirements of this study were explained to the subjects before the investigation. This survey was conducted anonymously, and the respondents filled out the questionnaires voluntarily. All participants gave their informed consent and deserve the right to withdraw from this study anytime. Inclusion criteria were as follows: at least 18 years of age; conforming to the diagnostic criteria of CS; having basic reading and writing skills. Patients having recently experienced emotional stress or a major life event; having cognitive disorder, or having other osteoarthropathies were excluded from this investigation. The study protocols were reviewed and approved by the Ethics Committee of Jinzhou Medical University and in accordance with the 1964 Helsinki declaration and its later amendments.

### Measures

#### Demographic questionnaire

Participants reported their age, sex, body mass index (BMI), marital status, education level, monthly income, job status, residence, smoking, drinking alcohol, family history, medical payment type, and whether having comorbid chronic diseases in this survey.

#### Measurement of resilience

Resilience level of patients was evaluated by the Resilience scale-14 (RS-14) (47). This scale has 14 items, including two dimensions: personal competence, and acceptance of self and life. A 7-point scoring ranging from strongly disagree to strongly agree was used for all the items. The RS-14 scoring ≤ 63, between 64 and 73, and ≥ 74 indicate being lowly, moderately, and highly resilient, respectively (13). The Chinese version of the RS-14 had strong reliability and validity (47, 48). In this study, Cronbach's α coefficient of the scale was 0.967.

#### Measurement of hope

The Herth Hope Index (HHI) was used in this study to evaluate hope (49). It consists of 12 items, each of which is rated from one to four. This scale measures three dimensions: temporality and future, positive readiness and expectancy, and interconnectedness. The higher HHI score means the higher hope level. HHI scale has been widely used in China, with high reliability and validity (32, 50). In the present study, Cronbach's α coefficient of internal consistency of the scale was 0.855.

#### Measurement of perceived social support

The Multidimensional Scale of Perceived Social Support (MSPSS) was used to assess an individual's satisfaction with their social support (51). The scale includes 12 items, all of which were rated on a 7-point Likert scale. MSPSS measures three different dimensions of perceived social support: family, friends, and significant others. The total score ranges from 12 to 84, with a higher total score indicating better social support. MSPSS exhibited good reliability and validity in China (52, 53). Cronbach's α coefficient of internal consistency of this scale was 0.943.

#### Measurement of optimism

The Revised Life Orientation Test (LOT-R) was used to measure the optimism level of the participants (54). The scale consists of six items, three of which measure optimism and three of which measure pessimism. All items were scored ranging from one to five. The items measuring pessimism were reversely scored, and thus the higher total score of the scale indicated higher level of optimism. The Chinese version of LOT-R exhibited satisfactory psychometric properties (55). In this study, Cronbach's α coefficient of the scale was 0.820.

#### Measurement of perceived stress

Perceived Stress Scale-10 (PSS-10) was used to measure the perceived stress of subjects (56). The scale contained 10 items in total. Each item was rated on a 5-Likert scale. The total scores ranged from 0 to 40, with higher score indicating higher level of perceived stress. PSS-10 showed good reliability and validity in China (57, 58). Cronbach's α coefficient of internal consistency of the scale was 0.820 in this study.

#### Measurement of anxiety symptoms

The Zung Self-Rating Anxiety Scale (SAS) (59) was used for measurement of anxiety, which included a total of 20 items. A 4-point scoring ranging from “never” to “always” was used for all the items. The total raw score was converted into a standardized int (1.25 × raw score), with higher scores indicating more severe anxiety symptoms. According to the cutoff value determined in the previous Chinese samples (60), anxiety was divided into three levels, which were mild (score 50–59), moderate (score 60–69) and severe (score ≥70). SAS showed good reliability and validity in China (61, 62). Cronbach's α coefficient of internal consistency of this scale was 0.884.

### Data analysis

Data was analyzed with SPSS version 20.0 and AMOS 22.0 (IBM Corporation, Armonk, NY, USA). Descriptive statistics with normal distribution were expressed by mean ± standard deviation (Mean ± SD). One-way ANOVA and student's *t*-test were used to analyze the differences of resilience in demographic variables. Exploratory factor analysis with Harman's single-factor test was used to address the concern of common method variation (CMV). Pearson's correlation was used to test the relationship between resilience, hope, social support, optimism, and perceived stress. Hierarchical regression analysis was used to determine the associated factors of resilience. Psychological factors were used as independent variables to determine predictors of resilience after inclusion of the statistically significant demographic variables in one-way ANOVA/*t*-test as control variables. Pearson's correlation was conducted to test the relationship between resilience, hope, social support, optimism, perceived stress, and anxiety. Hierarchical regression analysis was used to determine the associated factors of anxiety. A structural equation model was established to analyze the direct and indirect relationship on psychological variables. Bootstrap method was used to test the mediating effect of resilience on relationship between psychological variables and anxiety. Two-tailed probability value < 0.05 was considered statistically significant.

## Results

### Descriptive statistics

Sociodemographic characteristics of the subjects and the level of resilience in different demographic variables among CS patients were shown in Table 1. Of all the variables, the level of resilience proved to be significantly different among CS patients in different sociodemographic characteristics including marriage [*F*_(3, 246)_ = 10.515, *p* < 0.001], education [*F*_(3, 246)_ = 2.751, *p* = 0.043], job status [*F*_(3, 246)_ = 11.007, *p* < 0.001], monthly income [*t*_(248)_ = −6.616, *p* < 0.001], residence [*t*_(248)_ = 2.004, *p* = 0.046], family history [*t*_(248)_ = 5.511, *p* < 0.001], medical payment type [*t*_(248)_ = −4.937, *p* < 0.001], and whether having comorbid chronic disease [*t*_(248)_ = 6.889, *p* < 0.001].

**Table 1:** Demographic characteristics and the level of resilience among patients with cervical spondylosis (*n* = 250).

**Variables**	***N* (%)**	**Resilience**		
		**Mean (SD)**	***t*/*F***	**Cohen's d/η^2^**	***p***
**Age**			0.704	0.113	0.482
< 60 years	205 (82.0)	65.92 (15.92)			
≥60 years	45 (18.0)	64.04 (17.19)			
**Sex**			0.359	0.045	0.720
Male	121 (48.4)	65.96 (16.32)			
Female	129 (51.6)	65.22 (16.02)			
**Marriage**			10.515	0.114	< 0.001
Single	65 (26.0)	63.91 (16.94)			
Married	123 (49.2)	70.54 (13.88)			
Divorced	41 (16.4)	58.49 (16.07)			
Widowed	21 (8.4)	55.57 (16.21)			
**BMI**			1.537	0.012	0.217
< 18.5	21 (8.4)	59.76 (14.19)			
18.5–23.9	130 (52.0)	66.38 (15.66)			
≥24	99 (39.6)	65.77 (17.01)			
**Education**			2.751	0.033	0.043
Primary school	17 (6.8)	56.59 (14.23)			
Junior high school	84 (33.6)	65.01 (16.62)			
High school	70 (28.0)	65.11 (17.26)			
Junior college or above	79 (31.6)	68.53 (14.33)			
**Job status**			11.007	0.118	< 0.001
Employee	132 (52.8)	69.32 (15.86)			
Retirement	41 (16.4)	67.95 (15.82)			
Unemployed	18 (7.2)	51.17 (9.92)			
Other	59 (23.6)	59.97 (14.71)			
**Monthly income**			−6.616	−0.837	< 0.001
< 3,000	127 (50.8)	59.44 (14.63)			
≥3,000	123 (49.2)	71.92 (15.19)			
**Residence**			2.004	0.269	0.046
Urban	168 (67.2)	67.00 (15.99)			
Rural	82 (32.8)	62.67 (16.14)			
**Smoking**			0.852	0.108	0.395
No	128 (51.2)	66.43 (14.99)			
Yes	122 (48.8)	64.69 (17.28)			
**Drinking alcohol**			0.976	0.124	0.330
No	145 (58.0)	66.43 (15.20)			
Yes	105 (42.0)	64.41 (17.35)			
**Family history**			5.511	0.841	< 0.001
No	194 (77.6)	68.44 (15.34)			
Yes	56 (22.4)	55.68 (14.99)			
**Medical payment type**			−4.937	−0.893	< 0.001
Insurance	214 (85.6)	67.56 (15.45)			
Self-pay	36 (14.4)	53.83 (15.29)			
**Comorbid chronic disease (≥1)**			6.889	1.012	< 0.001
No	191 (76.4)	69.17 (14.63)			
Yes	59 (23.6)	53.97 (15.39)			

### Common method variance

According to exploratory factor analysis with Harman's single-factor test, a total of 14 factors had eigenvalues >1 when all the items of the scales used in the present study were included for analysis. The result showed that the first factor explained the maximum variance of 34.9%, suggesting an absence of common method variance in this study and the inter-relationship between these psychological variables can be effectively evaluated in the subsequent analysis.

### Correlation among resilience, optimism, perceived social support, hope, and perceived stress

Table 2 showed the correlation analysis results among resilience, optimism, perceived social support, hope, and perceived stress. Resilience was positively correlated with hope (*r* = 0.629, *p* < 0.001), perceived social support (*r* = 0.626, *p* < 0.001), optimism (*r* = 0.615, *p* < 0.001), and negatively with perceived stress (*r* = −0.651, *p* < 0.001).

**Table 2:** Descriptive statistics and correlations among continuous variables (*n* = 250).

**Variables**	**Means**	**SD**	**Resilience**	**Hope**	**Perceived social support**	**Optimism**
Resilience	65.58	16.14	1			
Hope	35.42	5.84	0.629^***^	1		
Perceived social support	57.75	11.68	0.626^***^	0.570^***^	1	
Optimism	22.96	4.30	0.615^***^	0.543^***^	0.469^***^	1
Perceived stress	15.50	6.26	−0.651^***^	−0.557^***^	−0.463^***^	−0.582^***^

*** *p* < 0.001.

### Hierarchical linear regression analysis of factors associated with resilience

Hierarchical linear regression analysis was used to explore the predictors of resilience among CS patients. Variables significantly correlated with resilience in the univariate analyses were included in the multiple regression model. Hierarchical linear regression analysis showed that hope (β = 0.145, *p* = 0.008), optimism (β = 0.226, *p* < 0.001), perceived social support (β = 0.223, *p* < 0.001), perceived stress (β = −0.229, *p* < 0.001), and whether having comorbid chronic disease (β = −0.131, *p* = 0.004) were significantly correlated with the resilience of CS patients, which could explain 63.9% of the total variance (Table 3).

**Table 3:** Hierarchical linear regression analysis on results of resilience (*n*= 250).

**Variables**	**Resilience**			
	**β**	***p***	**β**	***p***
**Step 1**				
**Marriage**				
Marriage_1	0.105	0.126	0.006	0.907
Marriage_2	−0.075	0.258	−0.054	0.273
Marriage_3	−0.017	0.790	−0.020	0.680
**Education**				
Education_1	0.073	0.496	0.027	0.732
Education_2	−0.001	0.991	0.008	0.919
Education_3	0.010	0.927	0.006	0.947
**Job status**				
Job status_1	0.312	0.006	0.091	0.286
Job status_2	0.236	0.010	0.116	0.088
Job status_3	0.095	0.322	0.004	0.951
Monthly income	0.212	0.001	0.038	0.433
Residence	0.015	0.807	−0.031	0.500
Family history	−0.143	0.014	−0.050	0.250
Medical payment type	0.136	0.017	0.048	0.258
Comorbid chronic disease	−0.192	0.002	−0.131	0.004
**Step 2**				
Hope			0.145	0.008
Perceived stress			−0.229	< 0.001
Optimism			0.226	< 0.001
Perceived social support			0.223	< 0.001
*F* value	9.775		25.523	
*p*-value	< 0.001		< 0.001	
*R* ^2^	0.368		0.665	
Adjusted *R*^2^	0.330		0.639	

Marriage_1 means Married vs. Single. Marriage_2 means Divorced vs. Single. Marriage_3 means Widowed vs. Single. Education_1 means Junior high school vs. Primary school. Education_2 means High school vs. Primary school. Education_3 means Junior college or above vs. Primary school. Job status_1 means Employee vs. Unemployed. Job status_2 means Retirement vs. Unemployed. Job status_3 means Other vs. Unemployed.

### Dyadic and comprehensive relations between anxiety and other psychological variables

Table 4 showed the correlation analysis results among anxiety, resilience, optimism, perceived social support, hope, and perceived stress. Anxiety was negatively correlated with resilience (*r* = −0.597, *p* < 0.001), hope (*r* = −0.589, *p* < 0.001), perceived social support (*r* = −0.443, *p* < 0.001), optimism (*r* = −0.549, *p* < 0.001), and positively with perceived stress (*r* = 0.599, *p* < 0.001).

**Table 4:** Dyadic relations between anxiety and other psychological variables.

**Variables**	**Resilience**	**Hope**	**Optimism**	**Perceived social support**	**Perceived stress**
Anxiety	−0.597^***^	−0.589^***^	−0.549^***^	−0.443^***^	0.599^***^

*** *p* < 0.001.

### Hierarchical linear regression analysis of factors associated with anxiety

In order to further explore the effects of resilience, hope, optimism, perceived social support and perceived stress on anxiety, multiple linear regression analysis was used to explore the associates of anxiety among CS patients. Multiple linear regression analysis showed that resilience (β = −0.181, *p* = 0.015), hope (β = −0.252, *p* < 0.001), optimism (β = −0.156, *p* = 0.013), and perceived stress (β = 0.251, *p* < 0.001) were the predictors of anxiety among CS patients, which could explain 48.0% of the total variance. However, there was no statistical significance for perceived social support (β = 0.003, *p* = 0.955) (Table 5).

**Table 5:** Multiple linear regression analysis on results of anxiety (*n* = 250).

**Variables**	**β**	***t***	***p***
Resilience	−0.181	−2.459	0.015
Hope	−0.252	−3.935	< 0.001
Perceived stress	0.251	3.907	< 0.001
Optimism	−0.156	−2.509	0.013
Perceived social support	0.003	0.057	0.955
*F* value	47.059		
*p*-value	< 0.001		
*R* ^2^	0.491		
Adjusted *R*^2^	0.480		

### The mediating role of resilience between other psychological variables and anxiety

**Figure 1:**
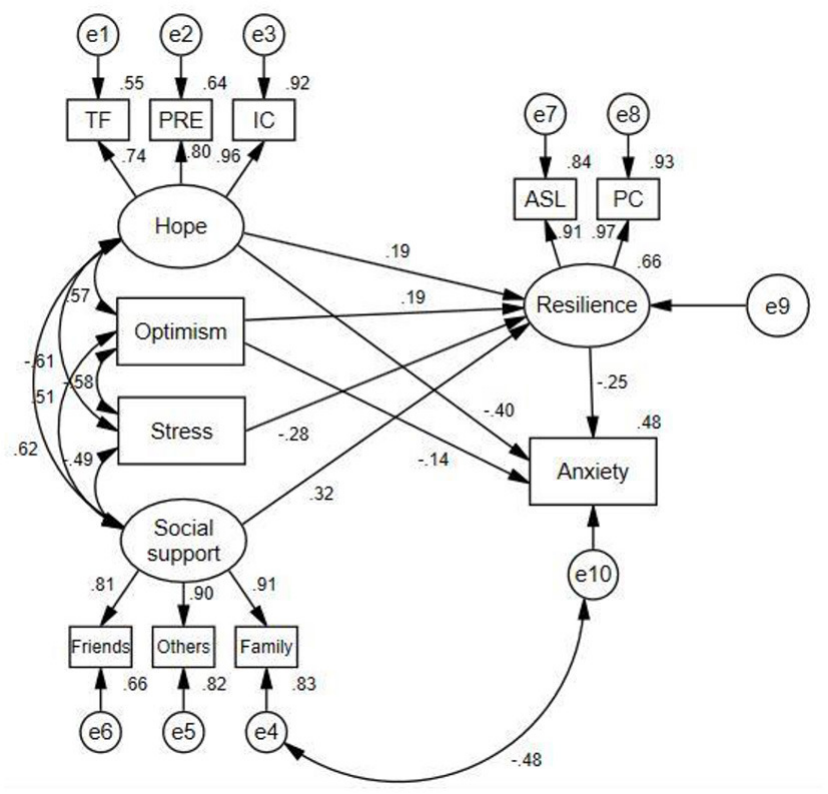
Path diagram for the hypothetical model. TF, temporality and future; PRE, positive readiness and expectancy; IC, interconnectedness; PC, personal competence; ASL, acceptance of self and life.

Structural equation model (SEM) was used to explore the relationship between variables, and to test the mediating role of resilience. The fit of the model was assessed by CMIN/DF, Goodness of Fit Index (GFI), Comparative Fit Index (CFI), Tucker Lewis Index (TLI), and root mean square error of approximation (RMSEA). After modification, the final model showed that the model fit well, CMIN/DF = 2.543, GFI = 0.940, CFI = 0.976, TLI = 0.960, RMSEA = 0.079. The final structural equation model was shown in Figure 1.

The path coefficients of hope on resilience and anxiety were 0.19 (*p* < 0.05) and −0.40 (*p* < 0.05), respectively. Optimism had a negative association with anxiety (β = −0.14, *p* < 0.05), and significantly positively predicted resilience (β = 0.19, *p* < 0.05). The path coefficients of perceived stress, and social support on resilience were −0.28 and 0.32 (both *p* < 0.05), respectively. Neither perceived stress nor social support had direct influence on anxiety.

The mediation effect was tested using bootstrap method with the bootstrap samples of 2,000 (Table 6). The results showed that resilience partially mediated the relationship between hope and anxiety among CS patients, and the mediating effect accounted for 10.3% of the total effect. We found that resilience partially mediated the effect of optimism on anxiety, with a mediating effect value of −0.048. Although social support and perceived stress had no direct effect on anxiety, they had indirect effect on anxiety through resilience, with mediating effect values of −0.080 and 0.070, respectively.

**Table 6:** The mediating effect of resilience between various psychological variables and anxiety.

**Variables**	**Effect**	**Point estimate**	**SE**	**Bootstrap**	
				**Bias-corrected 95% CI**	**Percentile 95% CI**
Hope	Total effect	−0.449	0.075	-0.600 to -0.312	-0.588 to -0.301
	Direct effect	−0.403	0.081	-0.570 to -0.256	-0.557 to -0.242
	Indirect effect	−0.046	0.023	-0.110 to -0.013	-0.097 to -0.008
Optimism	Total effect	−0.189	0.066	-0.306 to -0.047	-0.316 to -0.062
	Direct effect	−0.141	0.065	-0.266 to -0.006	-0.271 to -0.011
	Indirect effect	−0.048	0.023	-0.104 to -0.014	-0.097 to -0.010
Perceived stress	Indirect effect	0.070	0.034	0.020 to 0.156	0.018 to 0.150
Perceived social support	Indirect effect	−0.080	0.031	-0.159 to -0.031	-0.149 to -0.028

SE, standard error; CI, confidence interval.

## Discussion

The study was the first to investigate the resilience level and associated associates among CS patients, and to explore the impact of resilience-focused psychological factors on anxiety. Our results showed that the average value of resilience was 65.58 ± 16.14, indicating a moderate level of resilience. Among socio-demographic variables, marriage, education, job status, income, residence, family history, medical payment type, and whether having a chronic diseases were significantly associated with resilience. Our results also showed that hope, perceived social support, optimism were positively and perceived stress negatively associated with resilience. Hope, optimism, perceived social support, perceived stress, and whether having a chronic disease could be considered as independent associates with resilience among CS patients. Additionally, we found that anxiety was negatively (resilience, optimism, hope, perceived social support) or positively (perceived stress) correlated with the other five psychological variables in CS patients. The results of multiple linear regression showed that resilience, hope, optimism and perceived stress had significant effects on anxiety of CS patients. Finally, it was revealed that resilience was a mediator between various psychological factors and anxiety in CS patients, suggesting that improving resilience of CS patients by increasing hope, optimism, perceived social support and reducing the perceived stress would be an efficient and targeted treatment for the improvement of their anxiety status.

CS patients would frequently experience psychological stress including depression and anxiety (63). Mental resilience is an important and positive psychological resource to adaptively cope with adversity or unpleasant feelings (64). For example, we have previously shown that resilience would produce a significant influence on quality of life of patients with type 2 diabetes (65), and anxiety on patients with lumbar disc herniation (66). As shown in another instance, resilience was deemed as a protective factor against negative affect in dialysis patients (67). In spite of that, at present few studies are available regarding the resilience, its associates, and its influence on CS patient anxiety. This study presented evidence that the resilience of CS patients was at a moderate level, indicating that the resilience needs to be further improved in CS patients. Previously reported resilience training such as stress management and resiliency training (SMART) is expected to be efficient for resilience elevation in CS patients. This training method has been confirmed potentially feasible and effective in improving psychological well-being in women seeking preventive cardiology services (68), public school teachers and staff (69), and among new nurses (70). In addition to tentative application of exiting resilience inventions, such as SMART, more effective and targeted resilience intervention training need to be developed among CS patients, which requires a good understanding on the influencing factors of resilience in this affected population.

Resilience might be affected by various non-psychological and psychological factors (13, 65). In order to determine optimal resilience-based anxiety intervention strategies, great efforts were made in this work to find these contributors. We herein found resilience in CS patients was exclusively associated with whether having a comorbid chronic disease, among a host of other non-psychological/socio-demographic variables. Considering that resilience is a dynamic psychological adjustment process and might be impaired due to long-term and chronic unfavorable stimuli (71), one possible explanation was that CS patients with comorbid chronic diseases were more susceptible to physical and psychological tortures, resulting in decreased resilience and anxiety symptoms than those without chronic diseases (72). Therefore, more effort should be made to improve the resilience of those with comorbid chronic diseases. In addition to comorbid chronic diseases, our study also showed that resilience of CS patients was also influenced by multiple psychological factors, including hope, optimism, social support, and stress. The former two belonged to individual psychological qualities, and the latter two represented social environmental factors. Multivariable analysis results showed that these psychological attributes, together comorbid chronic conditions, explained as high as 63.9% variance of CS resilience. Intervention on these psychological variables would reasonably be regarded as effective and targeted treatment strategies for promoting resilience in CS patients.

Hope and optimism are important two constructs in positive psychology (15, 73). The positive effect of hope and optimism on resilience in CS patients as reported herein has also been found in other adversities (74). A recent study by Wu et al. found that self-report hope exerted a critical prediction on resilience in Chinese women newly diagnosed with breast cancer (74). Hope also predicts resilience following hereditary colorectal cancer genetic testing (75). Hope is an important inner psychological resource and could help individuals hold a positive outlook on life to mobilize resources to combat diverse adversities that they confront, playing a protection on individuals' mental health and well-being (76). A similar positive correlation between optimism and resilience was also corroborated by existing evidence (15). Studies have confirmed that optimism is a critical personality attribute connected with resilience (15). Of particular relevance to our present study, literature demonstrates that optimism is an important source of individual resilience and a protective mechanism against acute and chronic pain (77, 78). Optimistic individuals show higher capacities at emotional regulation to address negative conditions, tend to be receptive to any undesirable situations. Optimism can be deemed as a key personality trait to help maintain active engagement in effort to achieve a set goal even if in face of stressful situations (20). It is worth noting that whereas hope and optimism are commonly seen as personality traits, these two attributes are can also be learned and further developed during an individual's life. Previously optimism was proposed as an explanatory style (79). That is, an optimistic explanatory style interprets adversity or setbacks as temporary, whereas a positive event as enduring (78). As for hope, many intervention strategies have been suggested to increase its level (80–82), a recently reported short-intervention instrument in palliative cancer care, for example (80). For CS patients, incorporation of hope and optimism intervention strategies into resilience management might be helpful for improving their psychological distress.

Although there exists evidence showing that social support and stress were not statistically associated with resilience (13), our data supported that these two environmental arousals were indeed engaged in resilience regulation, which was consistent with other existing literature (83). Thus, although resilience is an inner strength or positivity and not easily affected by external stimuli, it can also be deemed as a changeable process due to constant individual-environmental interaction, which accords closely with the process definition of resilience construct (13). Social support enables individuals to adaptively change the way they interpret the events they are experiencing by engaging in cognitive restructuring and help increase self-regulation capacities when combating challenges or setbacks (84). Whereas, psychological stress generally affect one's cognitive processes, and impairs his/her psychological health, resulting in a range of psychopathological symptoms, such as isolation, rumination, and anxiety (83). As such, the correlation between social support and resilience (positive) and that between stress and resilience (negative) seems quite easy to understand. Interventions aimed at improving social support networks and decrease CS-related stress perception would be a reasonable option to increase patients' resilience and further decrease their anxiety level.

The mediation model analysis revealed that the effect of hope, optimism, social support, and stress on anxiety was partially or completely mediated by resilience. Thus, for CS patients, resilience seems derived from intra-personal psychological resources and perception on surrounding social environment. Similarly, a recent study also confirmed that perceived meaning in life, life satisfaction, and intolerance of uncertainty and hope played critical roles in resilience of Turkish adults during COVID-19 pandemic (85). Resilience can be regarded as a protective factor against psychological distress, including anxiety (10). Generally, resilient individuals can quickly adapt to the adversity in life, develop their potential to reduce the negative impact of disease, promote the recovery and development of patients' physical and mental functions, and further improve their quality of life (86, 87). Previously it was shown that SMART program was highly effective in reducing symptoms of stress, anxiety and depression (68). We also found that resilience was negatively correlated with anxiety among CS patients. This means that CS patients also benefit from mental resilience with regard to negative emotions, such as anxiety. Due to the absence of reports on the mental resilience of CS, our findings were meaningful for further understanding the resilience in CS patients. Based on our data, more attention should be paid to the exploration of intervention strategies aiming at improving mental resilience of CS patients, so as to develop their ability to cope with stress in the future.

This study identified the resilience of CS patients, its related factors, and comprehensive performance of these variables in anxiety among CS patients, which has important implications for the development of mental health intervention strategies. However, there are some limitations in methodology should be taken into consideration. First, due to the cross-sectional nature, causal inference cannot be made from the present survey results. Further longitudinal studies are needed to confirm our view. Secondly, self-reported psychological performance on hope, optimism, perceived social support, perceived stress, and anxiety would potentially bring about respondent bias. Thirdly, CS patients included in the current study were form the same hospital, thus the representativeness of the sample is a concern. A multi-center research is needed to confirm the generalizability of the conclusion as reported in the current study.

In conclusion, this study found that Chinese patients with CS presented a moderate level of mental resilience, which was independently and closely related to hope, optimism, social support, stress, as well as whether having comorbid chronic diseases. Resilience played a mediating role between various psychological variables and anxiety. Improving the level of resilience, hope, optimism and social support and reducing the level of stress are important strategies to reduce anxiety level. Relevant healthcare professionals should pay attention to the mental health of Chinese CS patients and help maintain good psychological status *via* improvement of resilience and its associated psychological variables.

## Data availability statement

The raw data supporting the conclusions of this article will be made available by the authors, without undue reservation.

## Ethics statement

The studies involving human participants were reviewed and approved by the Ethics Committee of Jinzhou Medical University. The patients/participants provided their written informed consent to participate in this study.

## Author contributions

YC and HD: conceived, designed the experiments, and wrote the paper. YC and YZ: data collection and methodology. YC and SW: analyzed and interpreted the data. All authors contributed to the article and approved the submitted version.

## Conflict of interest

The authors declare that the research was conducted in the absence of any commercial or financial relationships that could be construed as a potential conflict of interest.

## Publisher's note

All claims expressed in this article are solely those of the authors and do not necessarily represent those of their affiliated organizations, or those of the publisher, the editors and the reviewers. Any product that may be evaluated in this article, or claim that may be made by its manufacturer, is not guaranteed or endorsed by the publisher.
